# Unraveling the Molecular Basis of Mycosporine Biosynthesis in Fungi

**DOI:** 10.3390/ijms24065930

**Published:** 2023-03-21

**Authors:** Dionisia Sepúlveda, Sebastián Campusano, Martín Moliné, Salvador Barahona, Marcelo Baeza, Jennifer Alcaíno, Fernando Colabella, Blanca Urzúa, Diego Libkind, Víctor Cifuentes

**Affiliations:** 1 Departamento de Ciencias Ecológicas, Facultad de Ciencias, Universidad de Chile, Santiago 7800003, Chile; 2 Instituto Andino Patagónico de Tecnologías Biológicas y Geoambientales (Consejo Nacional de Investigaciones Científicas y Técnicas), CONICET-UNCo, Universidad Nacional del Comahue, Bariloche 8400, Rio Negro, Argentina; 3 Independent Researcher, Bariloche 8400, Rio Negro, Argentina; 4 Instituto de Investigación en Ciencias Odontológicas, Facultad de Odontología, Universidad de Chile, Santiago 8380492, Chile

**Keywords:** *Phaffia*, mycosporine, genes cluster, secondary metabolite

## Abstract

The *Phaffia rhodozyma* UCD 67-385 genome harbors a 7873 bp cluster containing *DDGS*, *OMT,* and *ATPG*, encoding 2-desmethy-4-deoxygadusol synthase, O-methyl transferase, and ATP-grasp ligase, respectively, of the mycosporine glutaminol (MG) biosynthesis pathway. Homozygous deletion mutants of the entire cluster, single-gene mutants, and the Δ*ddgs*^−/−^;Δ*omt*^−/−^ and Δ*omt*^−/−^;Δ*atpg*^−/−^ double-gene mutants did not produce mycosporines. However, Δ*atpg*^−/−^ accumulated the intermediate 4-deoxygadusol. Heterologous expression of the *DDGS* and *OMT* or *DDGS*, *OMT,* and *ATPG* cDNAs in *Saccharomyces cerevisiae* led to 4-deoxygadusol or MG production, respectively. Genetic integration of the complete cluster into the genome of the non-mycosporine-producing CBS 6938 wild-type strain resulted in a transgenic strain (CBS 6938_MYC) that produced MG and mycosporine glutaminol glucoside. These results indicate the function of *DDGS*, *OMT,* and *ATPG* in the mycosporine biosynthesis pathway. The transcription factor gene mutants Δ*mig1*^−/−^, Δcyc8^−/−^, and Δ*opi1*^−/−^ showed upregulation, Δ*rox1*^−/−^ and Δ*skn7*^−/−^ showed downregulation, and Δ*tup6*^−/−^ and Δ*yap6*^−/−^ showed no effect on mycosporinogenesis in glucose-containing medium. Finally, comparative analysis of the cluster sequences in several *P. rhodozyma* strains and the four newly described species of the genus showed the phylogenetic relationship of the *P. rhodozyma* strains and their differentiation from the other species of the genus *Phaffia*.

## 1. Introduction

Mycosporines, derived mainly from sedoheptulose-7-phosphate (SH-7P), are photoprotective compounds and products of the secondary metabolism of pro- and eukaryotic microorganisms [1,2,3]. They are intracellular, colorless, water-soluble molecules composed of a cyclohexenone core linked with one amino acid or amino-alcohol (MYCs) or an imine derivative with one or two such substituents (MAAs) [4,5,6]. These molecules exhibit an absorption spectrum between 310 and 360 nm [6,7] and have a photoprotective role against UV radiation (UVR), functioning as sunscreens [6,8]. In addition, they have other functions in the organisms that produce them, such as antioxidant and ROS scavenging, nitrogen storage, reproductive regulation and protection against osmotic stress, desiccation stress, and thermal stress [9,10,11,12]. Mycosporines have also been indicated to help wound healing and premature skin aging [13].

Mycosporines are widely distributed in nature and are found in bacteria, fungi, algae, and marine invertebrates, which have the capacity to synthesize them [1,14,15,16]. In addition, it has recently been reported that vertebrates can produce 4-deoxygadusol, a relevant component of mycosporines, de novo and that analogous pathways may be present in some amphibians, reptiles, and birds [17].

Fungi constitute an important group of mycosporine-producing microorganisms [18,19,20,21]. In addition to filamentous fungi, such as *Ascochyta fabae,* that produce mycosporine glutaminol glucoside (MGG) [22,23], the production of these molecules has also been described in yeasts, such as *Phaffia rhodozyma* (also known as *Xanthophyllomyces dendrorhous*) [12,16,20,24,25].

MYCs, unlike MAAs, have been less well studied, and following the discovery that the yeast *P. rhodozyma* also produces mycosporine glutaminol (MG) and mycosporine glutaminol glucoside (MGG) [16,26], several studies suggested that MGG production is an ancient (possibly plesiomorphic) trait in fungi [17]. This also indicates the existence of mycosporines and their photoprotective and antioxidant activity in yeasts [5], as well as their biotechnological potential [6,27].

Recently, it has been shown that most mycosporines produced by fungi are derived from SH-7P, an intermediate of the pentose phosphate pathway (PPP), in which a sugar phosphate cyclase (SPC), the 2-desmethyl-4-deoxygadusol synthase product of the *DDGS* gene [3,17], participates in the synthesis of 2-desmethyl-4-deoxygadusol (DDG). Previous studies suggested that the biosynthesis pathway for the MAA shinorine in *Anabaena variabilis* and *Nostoc punctiforme* consists of a core cluster of three genes encoding the enzymes Ddgs, Omt, and Atp-grasp, which catalyze the production of 2-desmethyl-4-deoxygadusol, which is followed by the formation of 4-deoxygadusol and the incorporation of glycine into the latter [1]. Finally, through the action of a nonribosomal peptide synthase (NRPS), serine is incorporated for the formation of shinorine [1]; the gene encoding this enzyme is part of the cluster. In *N. punctiforme*, however, the NRPS is replaced by the *Mys*D gene product to generate shinorine. In fungi, the cluster consists of the three core genes, *DDGS*, *OMT,* and *ATPG,* and lacks other genes encoding peptide-binding enzymes, producing only aminocyclohexenones, i.e., MYC and not MAAs [1]. Given that the cluster with the three genes *DDGS*, *OMT,* and *ATPG* is present in cyanobacteria as well as in fungi, that the genetic control of the biosynthesis pathway and the mechanisms underlying the regulation of this pathway in fungi are poorly understood [1], that the basidiomycete yeast *P. rhodozyma* produces MGG [15], and that candidate genetic elements were found in other strains of the species [28], a search was performed in the diploid strain UCD 67-385 of this species to identify mycosporinogenic genes. In this work, the structural genes that control the mycosporine biosynthesis pathway were identified in a cluster, and it was demonstrated that the production of this secondary metabolite in species of the genus *Phaffia* depends on the presence of these genes in the genome. This is the first report of the functionality of a mycosporine biosynthetic gene cluster in fungi.

## 2. Results and Discussion

### 2.1. Identification and Analysis of the Mycosporine Gene Cluster

Besides the very well-known ability of *P. rhodozyma* to synthesize the carotenoid astaxanthin as a product of secondary metabolism, recent research has shown that this species also produces MGG, another secondary metabolite that has a strong protective effect against UVR [5,6,7,8,9,10,11,12,13,14,15]. On the other hand, previous studies on the effect of glucose as a carbon source on carotenoid production have suggested that its synthesis is regulated by a catabolic repression process in which, in addition to Mig1, the Cyc8-Tup1 corepressor complex participates [29,30]. These proteins are the gene products of *CYC8* and *TUP1*, respectively. In a preliminary study to determine whether this complex plays an important role in the production of other secondary metabolites, such as mycosporines, we observed that the production of mycosporines in a homozygous Δ*cyc8*^−/−^ strain carrying a deletion mutation of the *CYC*8 gene was significantly higher than that in the wild-type strain of *P. rhodozyma* grown on medium supplemented with glucose as a carbon source.

**Figure 1:**
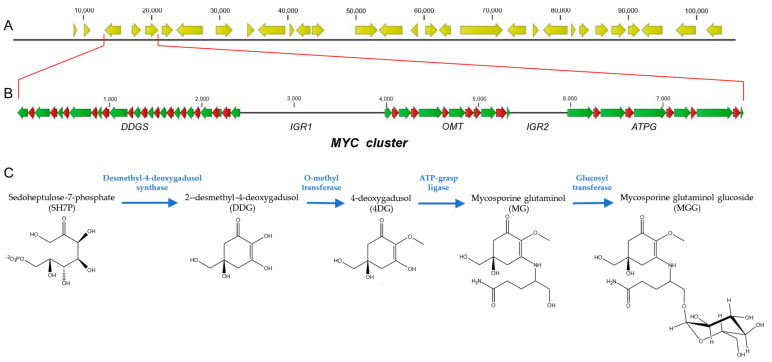
Graphical representations of the cluster of mycosporine genes from *P. rhodozyma* UCD 67-385. (**A**) A 100 kb region of the contig PacBio_000009 containing the MYC cluster at the left end. (**B**) Enlargement of the region containing the MYC cluster. The structural genetic organization of the *DDGS*, *OMT,* and *ATPG* genes is shown. (**C**) Mycosporine biosynthesis pathway. Green arrows: exons. Red arrows: introns. Thin black line: *IGR1* and *IGR2*.

Based on previous studies that demonstrated that *P. rhodozyma* produces mycosporines, specifically MGG [15], and that in fungi, the genes controlling mycosporine biosynthesis are organized on a cluster [1], we proceeded to search for the *DDGS*, *OMT,* and *ATPG* genes in the yeast genome of the diploid wild-type strain UCD 67-385 of *P. rhodozyma*. For this purpose, tBLASTn analysis was performed on the genome and transcriptome of this yeast strain, available in our laboratory [31], and the homologues of these genes found in several fungi were used. As a result, a 7873 bp cluster (MYC cluster) containing three genes involved in MGG biosynthesis was found in strain UCD 67-385. In fungi, secondary metabolite biosynthesis genes are commonly found in clusters [32], so it was expected that in *P. rhodozyma,* the genes controlling the mycosporine biosynthesis pathway would be organized in this manner, as has been observed in other microorganisms [1]. The MYC cluster was located at one end of a 1142 kb contig (PacBio_000009F). One of these genes in *P. rhodozyma* was first identified as encoding a dehydroquinate synthase (DHQS)-like protein. After comparative analysis of its deduced peptide sequence with those of cyanobacteria, bacteria, and fungi, a region between residues 269 and 299 containing the sequence **ML**D**LE**TG**NLHE**IK**LDRVIA**S**GH**TW**SP**IL**EL** was identified, suggesting that the enzyme is a fungal desmethyl-4-deoxygadusol synthase (Ddgs) and not a Dhqs [18]. Thus, the structural organization of the genes in this MYC cluster is *DDGS*—*OMT*—*ATPG*, similar to that in cyanobacteria. However, unlike that observed in cyanobacteria, the *DDGS* gene was encoded in the opposite direction from that of the *OMT* and *ATPG* genes (Figure 1). The sizes of the *DDGS*, *OMT,* and *ATPG* genes were 2414, 1361, and 1909 bp, respectively. Additionally, three sequences containing the open reading frames (ORFs) of the *DDGS*, *OMT,* and *ATPG* genes were identified from the transcriptome search. As expected, the ORFs colocalized in the MYC cluster at the same position and orientation as their respective genes. Comparative analysis of the genes and their transcripts allowed us to determine that *DDGS* contains 14 exons with an ORF of 1434 bp, corresponding to a protein of 477 amino acids with a deduced molecular mass of 52.6 kDa; the OMT gene contains seven exons with an ORF of 855 bp, corresponding to a 284-amino-acid protein with a molecular mass of 31.2 kDa; and the ATPG gene contains six exons with an ORF of 1503 bp, corresponding to a 500-amino-acid protein with a deduced molecular mass of 54.5 kDa (Figure 1). Additionally, BLASTp analysis of the respective sequences of each of the proteins revealed high levels of identity with corresponding proteins from other fungi. To characterize the product of each of the genes, they were expressed in *Escherichia coli*. For this purpose, the respective cDNAs were cloned, with a 6xHis-tag added at the amino terminus of each protein. The cDNA of the *DDGS* and *ATPG* genes was individually cloned into the pET TEV vector and expressed in *E. coli* strain BL21 (DE3). The *OMT* gene cDNA was cloned into the pGB1 fox p1 plasmid and expressed in *E. coli* strain Origami 2 (DE3). Protein analysis showed that the *DDGS*, *OMT*, and *ATPG* genes produced proteins with molecular masses of 54, 36, and 55 kDa, respectively, consistent with the masses of the deduced peptide sequences with the 6xHis-tag added (53.5, 32.1, and 55.5 kDa, respectively) (Figure S1).

On the other hand, in regard to the genetic organization of the MYC cluster, an intergenic region (*IGR1*) of 1568 bp was observed between the *DDGS* and *OMT* genes, which, since these genes are in opposite orientations, suggests that *IGR1* could share promoters that would act in a directional manner to their respective genes (Figure 1). In addition, between the *OMT* and *ATPG* genes, which have the same orientation, an intergenic region (*IGR2*) of 621 bp was observed, which would contain the promoter of the *ATPG* gene. The *IGR1* and *IGR2* intergenic regions were analyzed by searching for binding sites for transcription factors (TFs) with TFBSTools [33] and the JASPAR 2022 database [34]. After selecting TFs that interact with Cyc8 in *S. cerevisiae* and have a possible ortholog in *P. rhodozyma*, the analysis suggested that *IGR1* contained binding sites for the TFs Mig1, Skn7, Cup9, Rgt1, Rfx1, Sko1, Nrg1, and Rox1 and *IGR2* contained binding sites for the TFs Skn7, Cup9, Rfx1, Rgt1, Mig1, and Sko1.

Additionally, an RNA-seq comparative analysis of *CYC8* and *TUP1* gene deletion mutant strains grown in the presence of glucose for 36 h showed that the *DDGS* and *OMT* genes were overexpressed 5.7- and 2.9-fold in the Δ*cyc8*^−/−^ strain and 2- and 1.7-fold in the Δ*tup1*^−/−^ strain relative to the wild-type strain, respectively.

### 2.2. Determination of the Function of the MYC Cluster Genes in MGG Biosynthesis in Deletion Mutants

To demonstrate that the *DDGS*, *OMT*, and *ATPG* genes are involved in mycosporine biosynthesis in *P. rhodozyma*, homozygous deletion mutants of the entire MYC cluster and homozygous single mutants of each gene were constructed (Table S1, Figure S2). For this purpose, the diploid wild-type strain was transformed with a zeocin resistance module (Table S1C) that was integrated by a double crossover event in the outer adjacent sequence at both ends of the coding region, excluding the translation start and end codons of the gene of interest, replacing it and leading to the formation of a heterozygous zeocin-resistant deletion strain. The zeocin-resistant heterozygote was then transformed with a hygromycin resistance module (Table S1C), leading to the creation of a homozygous zeo^r^/hyg^r^ strain lacking both alleles of the gene of interest.

**Figure 2:**
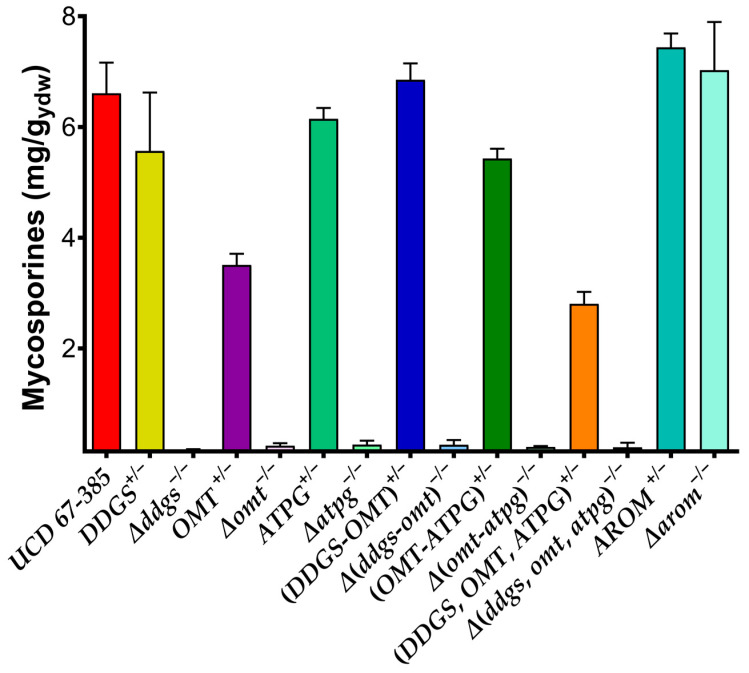
Phenotypic characterization of mycosporine mutant strains of *P. rhodozyma*. Mycosporine production of parental diploid wild-type UCD 67-385 and mutant strains were grown in YM complete medium with 1% glucose for 72 h. Error bars represent standard deviation.

The homozygous mutant strain Δ(*ddgs*,*omt*,*atpg*)^−/−^ contained a 7873 bp deletion spanning the entire cluster. The homozygous single mutants Δ*ddgs*^−/−^, Δ*omt*^−/−^, and Δ*atpg*^−/−^ contained deletions spanning the entire coding region of the respective genes. In addition, the double deletion mutants Δ(*ddgs-omt*)*^−/−^*, containing a 5343 bp deletion spanning both genes, and Δ(*omt*-*atpg*)^−/−^, possessing a 3891 bp deletion expanded from the *OMT* to the *ATPG* genes, were constructed. A homozygous mutant Δ*arom*^−/−^, containing a 5455 bp deletion spanning the entire *AROM* gene, was also constructed (Table S1A,B). The *AROM* gene encode a pentafunctional enzyme related to the aromatic amino acid biosynthesis. Single homozygous mutations of each gene, namely, Δ*ddgs*^−/−^, Δ*omt*^−/−^, and Δ*atpg*^−/−^, suppressed the production of mycosporines (Figure 2). However, the Δ*atpg*^−/−^ strain produced the intermediate 4-deoxygadusol. Analysis of the mycosporine production phenotype in a homozygous double mutant of the genotype *DDGS*^+/+^;Δ*omt*^−/−^;Δ*atpg*^−/−^ or in a double mutant of the genotype Δ*ddgs*^−/−^;Δ*omt*^−/−^;*ATPG*^+/+^, as expected, showed that mycosporines or their intermediates were not produced, regardless of the presence of the homozygous wild-type *DDGS*^+/+^ or *ATPG*^+/+^ gene, respectively. Similarly, the homozygous triple mutant strain Δ(*ddgs*,*omt*,*atpg*)^−/−^ did not produce mycosporines. Moreover, analysis of the mycosporine production phenotype in the heterozygous strains did not suggest the existence of a gene dosage effect on the biosynthesis pathway. Both the single and double heterozygotes (*DDGS*^+/−^;*OMT*^+/−^ and *OMT*^+/−^;*ATPG*^+/−^) exhibited a mycosporine production phenotype similar to that of the diploid wild-type parental strain, with the exception of *OMT*^+/−^, in which a slight deviation was observed, suggesting that the respective wild-type alleles exhibited dominance over the mutant alleles (Figure 2). However, this effect was not observed in the triple heterozygote *DDGS*^+/−^;*OMT*^+/−^;*ATPG*^+/−^, which produced half as much mycosporines as the wild-type homozygous parent, suggesting that the absence in cis of a complete copy of the MYC cluster led to incomplete dominance of the heterozygous strain, as reflected in its mycosporine production phenotype. Additionally, in the homozygous Δ*arom*^−/−^ mutant, the absence of the *AROM* gene, whose product has a Dhqs activity, exhibited a mycosporine production phenotype similar to that of the wild-type strain, a phenotype that was also observed in the heterozygous *AROM*^+/−^ condition. This suggests that in *P. rhodozyma,* there is no relationship between the Dhqs activity of the *AROM* gene product and MGG production and that the first stage of the biosynthesis pathway is catalyzed by the Ddgs enzyme from SH-7P to produce 2-desmethyl-4-deoxygadusol, which is converted into 4-deoxygadusol by the enzyme O-methyl transferase.

### 2.3. Heterologous Expression of DDGS, OMT, and ATPG P. rhodozyma Genes in S. cerevisiae

**Figure 3:**
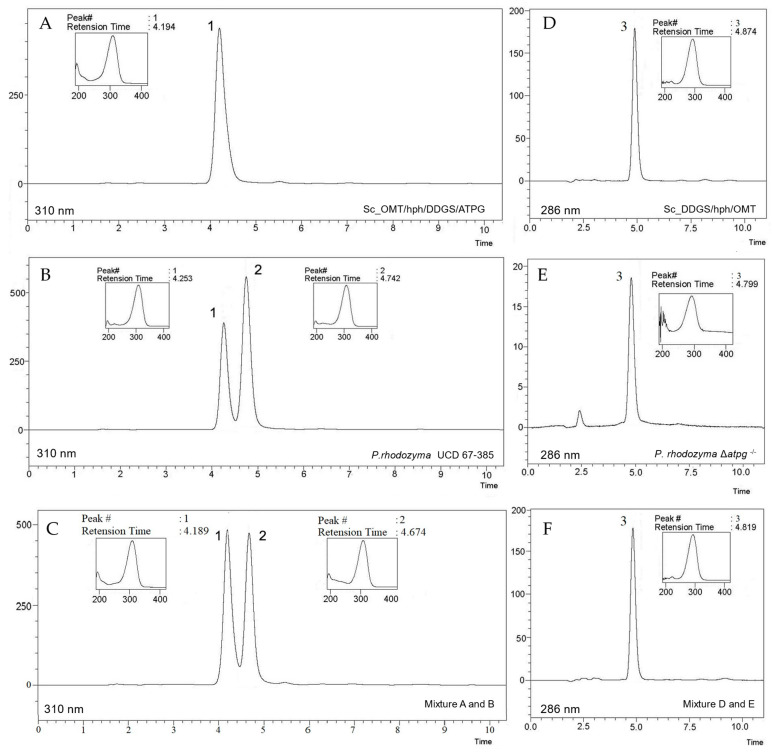
RP-HPLC analyses of mycosporines from *S. cerevisiae*. Representative chromatograms of mycosporines synthesized by heterologous expression of *P. rhodozyma* genes in *S. cerevisiae*. Peaks: 1 mycosporine glutaminol, 2 mycosporine glutaminol glucoside, 3 4-deoxygadusol. Samples from (**A**) Sc_OMT/hph/DDGS/ATPG, (**B**) *P. rhodozyma*, and (**C**) mixture of A and B. Samples from (**D**) 4DG extracted of Sc_DDGS/hph/OMT, (**E**) *P. rhodozyma* Δ*atpg*^−/−^, and (**F**) mixture of D and E. Insets represent the spectrophotometric scan of each peak.

In view of the previously confirmed role of the MYC cluster genes in mycosporine biosynthesis, we proceeded to study the heterologous expression of the three genes in the nonmycosporinogenic yeast *S. cerevisiae* S288C. For this purpose, two expression modules were used, one (OMT/hph/DDGS) containing the cDNAs of the *OMT* and *DDGS* genes from *P. rhodozyma* and the *hph* gene for hygromycin resistance from *E. coli* under the *TDH1*, *TEF1,* and *MET2* promoters and the *TEF1*, *TDH3,* and *CYC1* terminators of the *S. cerevisiae* transcript, respectively. The second module (OMT/hph/DDGS/ATPG) corresponded to the module (OMT/hph/DDGS) in which the cDNA of the *ATPG* gene from *P. rhodozyma* was incorporated under the *HIS3* promoter and *ADH2* terminator from *S. cerevisiae* downstream of the *DDGS* gene (Table S1C, Figure S3). A 750 bp segment from the start of the *LEU2* gene (Leu2up) located to the left of each expression module and the 345 bp end segment of the *LEU2* gene (Leu2dw) located to the right of each module were used as integration targets (Figure S3). Thus, the *S. cerevisiae* Sc_OMT/hph/DDGS/ATPG and Sc_OMT/hph/DDGS strains carried each respective module, which were inserted into the *LEU2* locus of chromosome III, disrupting it. Both strains were phenotypically auxotrophic for leucine and resistant to hygromycin (leu2^−^, hyg^r^). Analysis of the mycosporines synthesized by strain Sc_OMT/hph/DDGS/ATPG by HPLC chromatography showed a peak (Figure 3A, peak 1) corresponding to MG. Figure 3B shows the mycosporines produced by the wild-type strain of *P. rhodozyma*, with peak 1 corresponding to MG and peak 2 corresponding to MGG. Comparison of the retention times of peak 1 (corresponding to MG) of the Sc_OMT/hph/DDGS/ATPG strain (Figure 3A, peak 1) with peak 1 of the wild-type strain UCD 67-385 of *P. rhodozyma* (Figure 3B), which also corresponds to MG, showed that both were similar; however, the strains differed in the migration of peak 2, corresponding to MGG, which is only produced by *P. rhodozyma* (Figure 3B, peak 2). Furthermore, as expected, when the mycosporines produced by *S. cerevisiae* Sc_OMT/hph/DDGS/ATPG were mixed with those produced by *P. rhodozyma* and analyzed by HPLC, peak 1 of *S. cerevisiae* comigrated with peak 1 of the *P. rhodozyma* mycosporine sample (Figure 3C), confirming that the Sc_OMT/hph/DDGS/ATPG strain produced only MG.

Compared to previous results for the Δ*atpg*^−/−^ mutant of *P. rhodozyma*, analysis of mycosporine production in the Sc_OMT/hph/DDGS strain showed that it did not produce MG; it produced only the intermediate 4-deoxygadusol. In the HPLC analysis, the product synthesized by this strain corresponded to peak 3, with a retention time of 4.874 (Figure 3D). Comparison of peak 3 of the Sc_OMT/hph/DDGS sample with that observed for a sample from the Δ*atpg*^−/−^ strain of *P. rhodozyma*, which only produced 4-deoxygadusol (Figure 3E), showed that they both had the same retention time. Finally, both peaks comigrated when the products of the Sc_OMT/hph/DDGS strain were mixed with the products of the Δ*atpg*^−/−^ strain of *P. rhodozyma* (Figure 3F), indicating that the peak corresponded to 4-deoxygadusol.

The results obtained in the heterologous expression analyses were consistent with those observed with the products obtained with the MYC cluster gene mutations in *P. rhodozyma*, confirming the function of the *DDGS*, *OMT,* and *ATPG* genes in the MGG biosynthesis pathway of *P. rhodozyma*. Thus, the enzyme desmethyl-4-deoxygadusol synthase synthesizes the intermediate 2-desmethyl-4-deoxygadusol, which, through the action of the enzyme O-methyl transferase, leads to the biosynthesis of 4-deoxygadusol. The enzyme ATP-grasp ligase subsequently incorporates glutaminol into this intermediate molecule, leading to the formation of MG (Figure 1). A glucose molecule is subsequently incorporated into the MG molecule in *P. rhodozyma* to produce MGG, catalyzed by the product of a *P. rhodozyma*-specific gene that is not part of the MYC cluster and whose function is not present in *S. cerevisiae* (Figure 1).

### 2.4. Expression of the MYC Cluster in the Nonmycosporinogenic CBS 6938 Strain of P. rhodozyma

We examined several strains of *P. rhodozyma* from our laboratory collection and observed that eight isolates from Antarctica [35] and strain CBS 6938 did not produce mycosporines. In addition, genome analysis of three of these Antarctic strains shows that they lacked the mycosporine biosynthesis pathway gene cluster, which was corroborated by the absence of amplicons in PCR amplification assays for the *DDGS*, *OMT,* and *ATPG* genes.

In strain UCD 67-385, the MYC cluster (Genbank OQ547787) is located 14 kb from one end of the 1142 kb contig PacBio_000009F, the latter being equivalent to the 1107 kb contig Uchile_Xden1.PacBionly_10.1 of strain CBS 6938 (GenBank number JACGZH010000010.1). Comparative analysis of both contigs showed that strain CBS 6938 lacked a region of approximately 26.3 kb where the MYC cluster was located, consistent with the fact that it does not produce mycosporines.

**Figure 4:**
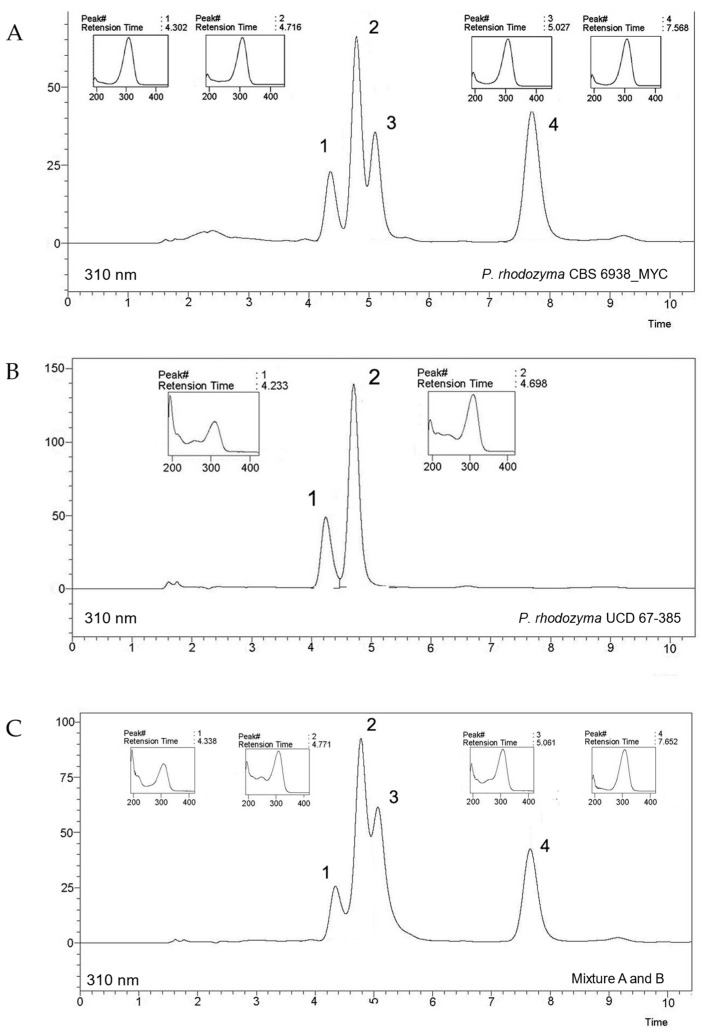
RP-HPLC analyses of mycosporines from the transformant CBS 6938_MYC strain of *P. rhodozyma*. Representative chromatograms of mycosporines synthesized by transgenic expression of mycosporine genes on the natural nonproducer strain CBS 6938 of *P. rhodozyma*. Chromatograms of mycosporines from the (**A**) transformant strain CBS 6938_MYC, (**B**) wild-type UCD 67-385 strain, and (**C**) mixture of (**A**) and (**B**) mycosporine samples. Insets represent the spectrophotometric scan of each peak.

To insert the MYC cluster into the contig JACGZH010000010.1 and demonstrate that the *DDGS*, *OMT,* and *ATPG* genes enable strain CBS 6938 to produce mycosporines, the plasmid pAss_Myc-3g-Hyg was constructed in vivo in *S. cerevisiae* by a DNA assembler [36,37]. For this purpose, eight DNA fragments were used whose ends overlapped, which allowed them, after transformation of *S. cerevisiae*, to be joined by homologous recombination in the designed order to generate the circular plasmid (Table S1G, Figure S4). The pAss_Myc-3g-Hyg plasmid was transferred into *E. coli* by transformation of the bacterium with total DNA purified from a *S. cerevisiae* Hyg^r^ transformant. Plasmid DNA was purified from the bacterium and digested with *Xba*I to release the Ass_Myc-3g-Hyg module with which *P. rhodozyma* strain CBS 6938 was transformed, yielding 21 hygromycin-resistant transformants (Figure S5). PCR analysis was performed to determine the presence of the MYC cluster in six hygromycin-resistant transformants producing mycosporines. All six transformants carried the mycosporine biosynthesis genes and exhibited the mycosporine production phenotype and hygromycin resistance. A transformant (CBS 6938_MYC) was selected from which mycosporines were extracted and analyzed by HPLC. The strain CBS 6938_MYC produced four peaks, of which peaks 1 and 2 corresponded to MG and MGG (Figure 4A). Peaks 3 and 4 correspond to unidentified products. Both peaks 1 and 2 corresponded to those observed in the chromatogram of the wild-type diploid strain UCD 67-385 (Figure 4B). The chromatographic profile of a mixture of the mycosporines produced by strains CBS 6938_MYC and UCD 67-385 showed that peaks 1 and 2 overlapped, indicating that strain CBS 6938_MYC synthesizes MG and MGG (Figure 4C).

**Figure 5:**
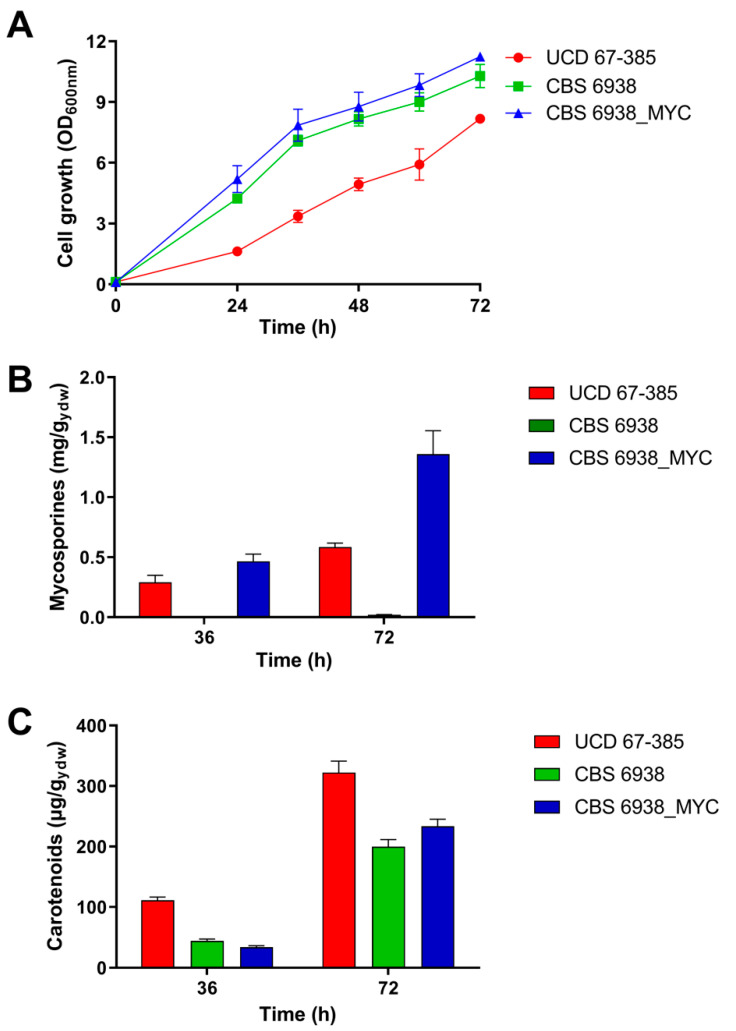
Phenotypic characterization of the transformant CBS 6938_MYC strain of *P. rhodozyma*. (**A**) Growth profile of the donor UCD 67-385, receptor CBS 6938 (wild type) and transformant CBS 6938_MYC. (**B**) Mycosporine production. (**C**) Carotenoid production. The strains were grown in 1% glucose-containing YM medium. Error bars represent standard deviation.

Analysis of growth on YM medium supplemented with 1% glucose showed that the non-mycosporine-producing wild-type parental strain CBS 6938, lacking the MYC cluster, and the mycosporine-producing strain CBS 6938_MYC were similar. However, both strains differed from the wild-type strain UCD 67-385, a donor of the MYC cluster and a natural producer of mycosporines, which showed less development during its entire culture period (Figure 5A). Since both strains CBS 6938 and CBS 6938_MYC possessed the same genetic background with the exception that the latter carried the module containing the MYC cluster and included a hygromycin resistance gene, the acquisition of the additional genetic material and the ability to produce MG and MGG had no discernible effect on their growth characteristics. Analysis of mycosporine production showed that mycosporine biosynthesis in the transgenic strain was higher than that in the UCD 67-385 donor strain at both 36 and 72 h of culture, with the yield increasing 1.4- and 2.6-fold, respectively (Figure 5B). The high yield observed in strain CBS 6938_MYC suggests that the CBS 6938 genetic background has a higher potential to express cluster genes and produce larger amounts of mycosporines than the UCD 67-385 donor strain without additional genetic modifications. These results suggest that the parental strain CBS 6938 could be a more suitable host for the development of breeding programs to increase its performance in industrial production. Additionally, analysis of the production of another secondary metabolite, astaxanthin, showed that the wild-type strain UCD 67-385 produced more of this pigment than the strains CBS 6938 and CBS 6938_MYC. Furthermore, the CBS strains did not differ significantly from each other in carotenoid production at both 36 and 72 h of culture (Figure 5C). Both types of secondary metabolites, mycosporines and carotenoids, are produced in the late growth stage of *P. rhodozyma*, when the carbon source has been or is close to being fully consumed. This suggests that glucose could have a regulatory effect on this pathway. In the case of astaxanthin biosynthesis, it has been observed that mutations in the *MIG1*, *CYC8,* and *TUP1* genes lead to an increase in carotenoid production and could be involved in the regulation of catabolic glucose repression [29,30,38]. In addition, it has been observed that the products of the *CYC8* and *TUP1* genes constitute a complex (Cyc8-Tup1) that interacts with different TFs, modifying the expression of other structural genes [38,39,40].

### 2.5. Expression Analysis of Mycosporine-Related Metabolic Pathways in Mutant Strains of Transcription Factor Genes of P. rhodozyma

Mycosporine production has been associated with yeast survival under UV stress [15] and other types of stress (osmotic, desiccation, oxidative, etc.) Recognition sequences of TFs involved in the stress response were observed in the *IGR1* and *IGR2* regions of the MYC cluster, and mycosporine production was measured in deletion mutants of the *ROX1*, *SKN7*, *YAP6*, and *OPI1* genes. Mycosporine production was also investigated in deletion mutants of the *MIG1*, *CYC8*, and *TUP1* genes, which, in *S. cerevisiae*, participate in glucose repression through the Cyc8-Tup1 complex [41], and in *P. rhodozyma*, participate in the regulation of the production of secondary metabolites such as astaxanthin [29,30] and mycosporines [40].

**Figure 6:**
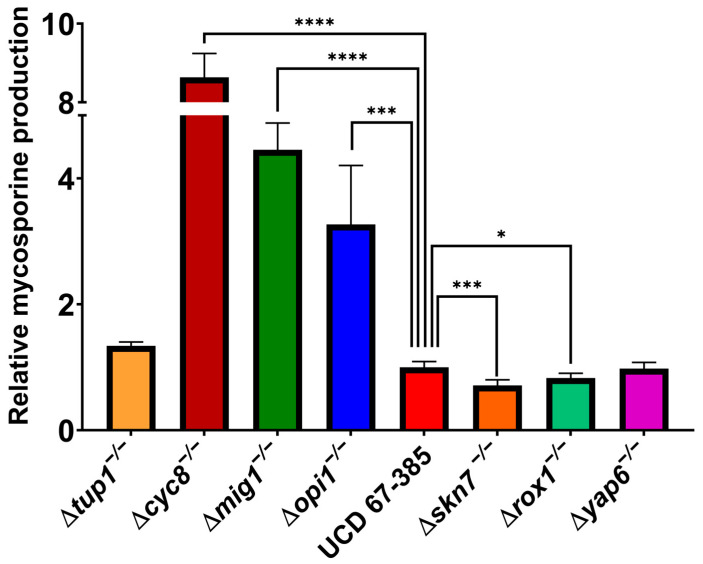
Mycosporine production of the transcription factor gene mutants relative to the parental *P. rhodozyma* strain UCD 67-385. The strains were grown in YNB medium containing 2% glucose. One-way ANOVA followed by Fisher’s LSD test (* *p*-value < 0.05, *** *p*-value < 0.001; **** *p*-value < 0.0001). Error bars represent standard deviation.

Homozygous deletion mutants Δ*mig1*^−/−^, Δ*cyc8*^−/−^, and Δ*opi1*^−/−^ of *P. rhodozyma* overproduced mycosporines, and the mutants Δ*rox1*^−/−^ and Δ*skn7*^−/−^ produced less mycosporines than the wild-type strain in medium supplemented with glucose (Figure 6). In the strains Δ*tup1*^−/−^ and Δ*yap6*^−/−^, no significant differences in mycosporine production were observed with respect to the wild-type strain. These results suggest that in mycosporine biosynthesis, the *SKN7* and *ROX1* gene products participate by positively regulating the expression of mycosporinogenic genes. In *S. cerevisiae*, Skn7 interacts with the Cyc8–Tup1 complex, recruiting Tup1 for optimal induction of genes for oxidative stress response, osmoregulation, hypoxia response and induction of heat shock proteins in response to oxidative stress [41,42,43]. In addition, an RNA-seq comparative analysis of *CYC8* and *TUP1* gene deletion mutant strains was carried out based on previous results [39,40]. This analysis showed that the expression levels of the messengers of the *DDGS* and *OMT* genes were 5.7- and 2.9-fold, and 2- and 1.7-fold higher, respectively, than those in the wild-type parental strain.

**Figure 7:**
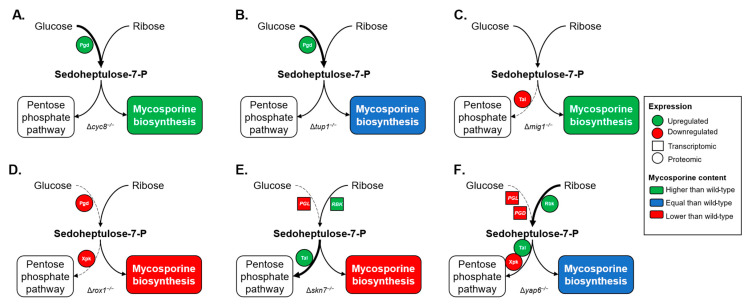
Effects of the *CYC8*, *TUP1*, *MIG1*, *ROX1*, *SKN7,* and *YAP6* gene deletions in *P. rhodozyma* on the pentose phosphate pathway and their correlation with mycosporine biosynthesis. Differentially abundant proteins from proteomic studies and differentially expressed genes from transcriptomic studies were mapped to the pentose phosphate pathway, emphasizing effects on enzymes that may affect the availability of sedoheptulose-7-P for the production of mycosporines. The strains Δ*cyc8*^−/−^ (**A**), Δ*tup1*^−/−^ (**B**), Δ*mig1*^−/−^ (**C**), Δ*rox1*^−/−^ (**D**), Δ*skn7*^−/−^ (**E**), and Δ*yap6*^−/−^ (**F**) were grown on YNB-glucose minimal medium and compared to the wild-type strain. The proteomic data are represented by filled circles, and transcriptomic data are represented by filled squares. Thick and dashed arrows represent the proposed metabolic flux based on the expression profile. Enzymes, genes: 6-phosphogluconolactonase (Pgl, *PGL*), 6-phosphogluconate dehydrogenase (Pgd, *PGD*), ribokinase (Rbk, *RBK*), transaldolase (Tal, *TAL*), xylulose-5-P phosphoketolase (Xpk, *XPK*).

To determine the biological processes that may account for the changes in mycosporine production observed, RNA-seq and proteomic data from previous studies were analyzed [39,40], focusing on mycosporine-related pathways (Table S2). The enzyme 6-phosphogluconate dehydrogenase (Pgd) was more abundant in the Δ*cyc8*^−/−^ mutant strain than in the wild-type strain, and the *DDGS* gene was upregulated (Figure 7A). Since Pgd catalyzes the formation of ribulose-5-P, a precursor of SH-7P in the PPP, these observations were consistent with the higher mycosporine production observed in this strain. Pgd was also more abundant in the Δ*tup1*^−/−^ strain (Figure 7B), but no effect on mycosporine content was observed. On the other hand, Pgd abundance was lower in the Δ*rox1*^−/−^ mutant strain (Figure 7D), which was consistent with the lower mycosporine production in this strain than in the wild-type strain. The enzyme transaldolase (Tal) was less abundant in the Δ*mig1*^−/−^ mutant strain than in the wild-type strain (Figure 7C). This enzyme uses SH-7P as a substrate to form other compounds in the PPP; thus, lower abundance of this enzyme leads to increased mycosporine accumulation. Meanwhile, the Δ*skn7*^−/−^ mutant strain showed a higher abundance of Tal than the wild-type strain (Figure 7E), which was also consistent with lower mycosporine production. Interestingly, the *DDGS* gene was upregulated in this strain, but the mycosporine content was still lower than that in the wild type. The enzyme 6-phosphogluconolactonase (Pgl) catalyzes the formation of gluconate-6-P, which is a substrate used by Pgd to form ribulose-5-P. In the Δ*skn7*^−/−^ mutant strain, the expression of the *Pgl* gene was downregulated (Figure 7E), which was also consistent with the mycosporine content observed. Furthermore, enzymes such as Tal and ribokinase (Rbk) were more abundant in the Δ*yap6*^−/−^ mutant strain than in the wild-type strain, while Pgl and xylulose-5-P phosphoketolase (Xpk) were less abundant (Figure 7F). Additionally, the *OMT* gene was downregulated in this strain, but mycosporine production in this strain was not significantly different compared to that in the wild-type strain. Rbk phosphorylates ribose to form ribose-5-P, a precursor of sedoheptulose-7-P, and Xpk uses xylulose-5-P as a substrate, another precursor of SH-7P, to form other compounds in the PPP. A high abundance of Rbk and a low abundance of Xpk may suggest higher mycosporine production, but a high abundance of Tal and a low abundance of Pgl may be related to lower mycosporine production. Since these events occurred simultaneously in this strain, an equal mycosporine content was expected.

In view of the above, the genetic control of metabolism and gene physiology would provide a mechanism with a high level of genetic plasticity. *P. rhodozyma* lives in a natural habitat with varied characteristics, including low temperatures, windy conditions, high altitudes, exposure to intense light and UVR, and high levels of oxidant compounds [44,45]. Regulation of the metabolic pathways enables yeast cells to respond to environmental conditions and the presence of damaging factors through the expression of genes controlling the biosynthesis of astaxanthin and mycosporines. Early studies indicated that this basidiomycete yeast species has low catalase activity [46], and two superoxide dismutases have been identified in its genome [28], which are common antioxidant systems present in living organisms to protect them from H_2_O_2_ or other reactive oxygen species that produce photooxidative damage. These protection mechanisms are necessary, but not sufficient, for survival on leaves, tree barks, or tree exudates in mountainous and cold regions in Southern Chile and Argentina [35,47,48] or other places, such as Alaska, Russia, or Greenland [49,50,51]. As a product of their metabolism, leaves exude chemical compounds that may react with ozone or with the intense light or UVR that they are exposed to in their environment, generating free radicals, ROS, and other harmful products to the yeast [46,52,53]. Similarly, other living organisms, including microbes, share the same habitat, and these organisms may produce polyols, causing oxidative and osmotic stress, among other stresses. Additionally, wounds are generated on tree trunks from which sugar-, polyol-, and salt-rich exudates are produced. Chemical compounds in these exudates may also react with intense light or UVR due to ozone layer limitations. In all these ways, compounds harmful to the cells may also change the osmolarity of the medium and cause stress. Cold and strong winds contribute to the desiccation of the habitat and, therefore, of *P. rhodozyma* cells, causing damage that must be repaired. Additionally, UVR and other metabolites damage the cell wall, membrane, and genetic material, which must then be repaired. At high altitudes, the concentration of oxygen is lower, causing hypoxia, which affects the general metabolism of yeast. Due to all of these events, *P. rhodozyma* must protect itself from damage, for which is has evolved several mechanisms. To this end, secondary metabolites play an important protective role. These include astaxanthin, a carotenoid with powerful antioxidant activity and a high photoprotective capacity, and MGG, a mycosporine that provides protection against intense light, UVR, and other stresses, such as oxidative, osmotic, desiccation, and cold stress [4,5,15,45]. It is possible that different mechanisms of gene expression regulation are induced under these conditions. Most of them could be related to the Cyc8–Tup1 complex. Depending on the stress, the Cyc8–Tup1 complex interacts with alternative TFs or regulators such as Skn7, Rox1, Yap6, and Opi1 so that the yeast can respond to different types of stress by repressing or activating gene expression.

### 2.6. MYC Cluster in Different Species of the Genus Phaffia

**Figure 8:**
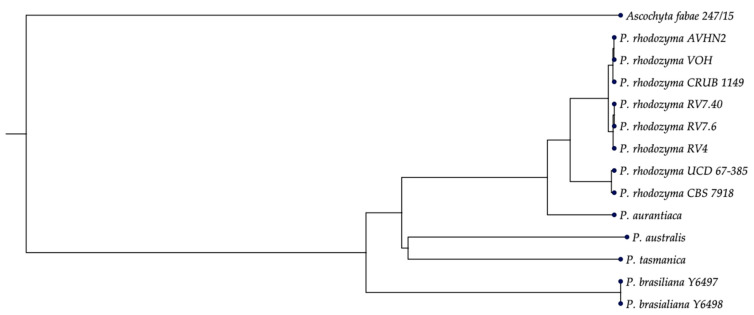
Phylogenetic placement of *Phaffia* species based on a combined alignment of the MYC cluster containing the structural genes controlling the mycosporine biosynthesis pathway and the two regulatory intergenic regions, *IGR1*/*IGR2*. Maximum Likelihood Phylogeny 1.3. UPGMA, Kimura 80 model construction, and bootstrap analysis with 1000 replicates were performed with CLC Genomics Workbench 22.0.2 software. *Ascochyta fabae* was used to root the tree.

The genus *Phaffia* was initially described with a single species, *Phaffia rhodozyma* [49], which has many strains isolated from different geographical locations, including the Antarctic, obtained from water samples in lakes, soils, exudates, leaves, and the bark of tree trunks. However, in the last three years, at least four new species have been described, namely, *P. australis*, *P. tasmanica*, *P. brasiliana*, and *P. aurantiaca*, transforming from a monospecific to a penta-specific genus [54,55,56]. All species of the genus *Phaffia* naturally synthesize astaxanthin as a major carotenoid and can produce MGG. However, not all strains of *P. rhodozyma* produce mycosporines, for which there is a genetic explanation, i.e., the absence of the MYC cluster, which carries the *DDGS*, *OMT,* and *ATPG* genes, responsible for the mycosporine production phenotype. All strains of *P. rhodozyma* and species of the genus *Phaffia* studied in this work that produce mycosporines contain the MYC cluster in their genome. In addition, the six genes required for astaxanthin biosynthesis in *Phaffia* represent a typical genetic hallmark of the genus [54]. Furthermore, in the astaxanthin biosynthesis pathway, the conversion of beta-carotene to astaxanthin is catalyzed by the enzyme astaxanthin synthase [57,58]. This, in a strict sense, is not evolutionarily a carotenogenic gene but converges in the carotenogenesis of yeast from a P450 monooxygenase that requires a cytochrome P450 reductase product of the *CRTR* gene [59]. These conditions give the genus *Phaffia* a particular characteristic, as it makes it the only carotenogenic yeast with this functional genetic organization for the genetic control of the production of this secondary metabolite. Based on the above, a comparative analysis was performed using only the nucleotide sequences of the MYC cluster, including the regulatory regions *IGR1* and *IGR2*, in the genomes of *Phaffia* available in GenBank and in our laboratory (Table S1H). For all analyzed strains the synteny and orientation of the genes of the MYC cluster are similar indicating that they are relatively conserved within the genus *Phaffia*. This allowed us to run a phylogenetic analysis with all *Phaffia* spp. strains with available MYC cluster sequences. The results, shown in Figure 8, indicated that the phylogenetic structure obtained with the MYC cluster DNA sequences was consistent with the results obtained from phylogenomic analyses using amino acid sequences of 471–485 orthologous single-copy genes [54,55]. This means that the genes are orthologous and derive from a single evolutionary event. Thus, the presence of the mycosporine gene cluster and the production of mycosporines is observed in all species of the genus *Phaffia,* albeit a few strains of *P. rhodozyma* naturally lack the gene cluster and thus the ability to synthesize MGG. Whether this occurs in strains of the other species of the genus it is still unknown given only one or at most two strains of each species have genomes available for analysis. All South American isolates from Argentina and Chile clustered together closely related to the European lineage that contains the strain CBS 7918T, similar to what was previously seen using rDNA loci and genome based phylogenies [55]. *P. aurantiaca* position is typically placed next to both South American and European lineages as shown previously [55]. The Australasian species *P. australis* and *P. tasmanica* were clustered together in this analysis, which differs from previous studies [54,55]. These results showed that the MYC cluster may be a potential molecular marker for phylogenetic analyses of mycosporine-producing fungi.

*P. rhodozyma* is the oldest and best-known species of the genus, and many strains of this species have been isolated in different environments on Earth. Natural strains that do not produce mycosporines have also been observed, and this phenotype correlates with the lack of the MYC cluster in these strains. Mutant strains lacking MYC in *P. rhodozyma* were significantly less tolerant to UVB exposure than their wild counterparts [60]. Similar results were obtained with other yeast species [5] suggesting that this trait has an ecological value in solar irradiated substrates for yeasts. On the other hand, MGG is a biotechnologically important molecule due to its antioxidant and UV sunscreen activities [26], and the high intracellular concentrations that *P. rhodozyma* achieves in batch cultures [15]. Hence, the present work has helped to unravel the molecular basis of mycosporine synthesis in fungi, a very important feature of both fundamental and applied relevance.

## 3. Materials and Methods

### 3.1. Strains and Culture Conditions

*P. rhodozyma* strains (Table S1A in the supplemental material) were grown at 22 °C with constant shaking in a 1 L Erlenmeyer flask containing 380 mL of 0.7% YNB minimal medium (YNB DIFCO BD 291940) supplemented with 2% glucose. When necessary, the strains were grown in YM medium (1% glucose, 0.3% yeast extract, 0.3% malt extract, and 0.5% peptone). The cells were grown to the early exponential growth phase (OD600 of 1.6–2.5) and then collected for phenotypic characterization. Optical density was measured at 600 nm using a JASCO V-630 spectrophotometer (JASCO Inc., Easton, MD, USA). Samples were harvested through centrifugation, and the pellet was washed twice with ice-cold water, centrifuged at 5000× *g* for 10 min at 4 °C, and stored at −80 °C until further analysis.

*P. rhodozyma* mutant strains for the genes *DDGS, OMT, ATPG,* and *AROM* were derived from the wild-type strain UCD 67-385 (ATCC 24230) and were obtained via homologous recombination to replace the corresponding gene with a cassette that confers resistance to an antibiotic [61] (see Table S1A–D).

The *S. cerevisiae* strains were cultured at 30 °C in YPD medium. When necessary, the transformant strains were grown in YPD medium supplemented with 200 μg/mL hygromycin B.

The *E. coli* strains were cultured with constant shaking at 37 °C in LB medium. The LB agar plate medium was supplemented with 100 μg/mL ampicillin for the selection of recombinant clones.

### 3.2. Identification of the DDGS, OMT, and ATPG Genes of P. rhodozyma

The *DDGS, OMT,* and *ATPG* genes of *P. rhodozyma* were identified through homology searches using tblastn on CLC Genomics Workbench. Sequences of DHQS-homolog proteins from *P. tritici*-*repentis* (EDU45310) and *P. nodorum* (EAT88778), O-methyl transferase from *A. clavatus* (EAW13536), and *M. orzyae* 70-15 (EDK03437) and ATP-grasp from *P. tritici-repentis* (EDU45308) and *P. nodorum* (EAT88781) were searched against the genome and transcriptome sequences of the strain UCD 67-385 of *P. rhodozyma* available in our laboratory [31]. Transcripts with the best e-value were selected as orthologs of each gene. Graphical representations of genes and gene products were carried out on CLC Genomics Workbench 22.02 software.

The *IGR1* and *IGR2* genomic regions of MYC cluster were analyzed with TFBSTools 1.34.0 [33] and JASPAR2022 0.99.7 [34] in R version 4.2.0. The search was limited to “fungi” tax group and only the latest version of each Position Frequency Matrix (PFM) was used. Relative score cutoff of 80% was used, and both strands of each sequence were analyzed. The matrix ID analyzed were MA0288.1 (Cup9), MA0337.1 (Mig1), MA0347.2 (Nrg1), MA0365.1 (Rfx1), MA0367.1 (Rgt1), MA0371.1 (Rox1), MA0381.1 (Skn7), and MA0382.2 (Sko1).

### 3.3. Phenotypic Determination

#### 3.3.1. Pigment Extraction

Pigments were extracted from cellular pellets by acetone extraction [40]. In brief, aliquots of cell cultures were collected under the different conditions tested. The aliquots were centrifuged at 4000× *g*, and the supernatants were subsequently discarded. Each cell pellet was suspended in 2 mL of an acetone:water mixture (1:1), and 500 μL of 0.5-mm glass beads was then added. After 3 min of vortex shaking, the mixture was centrifuged at 4000× *g* for 5 min. Next, the supernatant was transferred to a clean test tube, and 2 mL of acetone was added to the pellet. The tube containing the pellet was then vortexed, stirred for 3 min, and centrifuged at 4000× *g* for 3 min, after which the supernatant was collected and mixed with the supernatant that had been previously set aside. These steps were repeated until the recovered supernatant was completely colorless.

The collected supernatants were then treated with 0.25 volumes of water and 0.25 volumes of petroleum ether; this mixture was mixed and centrifuged for 5 min at 4000× *g*. Subsequently, the petroleum ether (top) phase was recovered, its absorbance at 474 nm was measured, and the carotenoid concentration was estimated using an absorption coefficient of A1% = 2100. It was normalized to the yeast dry weight. The analyses were performed in triplicate.

#### 3.3.2. Mycosporine Extraction

Mycosporines were extracted from cell pellets by resuspension in 1 mL of 80% ethanol, incubated for 2 h at 80 °C, and then centrifuged at 14,000 rpm for 2 min. In the recovered supernatant, the mycosporine concentration was spectrophotometrically determined at 310 nm using the molar extinction coefficient E_m_ = 25,000 M^−1^ cm^−1^ [40] and normalized by the dry weight of the yeast. The extracted mycosporines were separated by RP–HPLC using a C-18 Lichrocart 125-4 column (Merck, Rahway, NJ, USA) with H_2_O:methanol:acetic acid (99.3:0.5:0.02, *v*/*v*/*v*) and H_2_O:methanol (97:3, *v*/*v*) as the mobile phases and a 1 mL/min flux under isocratic conditions. Mycosporines were identified according to their retention time and absorption spectra.

### 3.4. Statistical Analyses

The statistical analyses were performed using GraphPad Prism version 9.3.1 (GraphPad Software, San Diego, CA, USA). No mathematical correction was made for multiple comparisons, and all comparisons performed were planned and reported in this work.

### 3.5. RNA-Seq and Proteomic Data Analysis

Expression profiles from the Δ*cyc8*^−/−^, Δ*tup1*^−/−^, Δ*mig1*^−/−^, Δ*rox1*^−/−^, Δ*skn7*^−/−^, and Δ*yap6*^−/−^ mutant strains from previous studies were reanalyzed. Transcriptomic data from RNA-seq experiments and proteomic data from iTRAQ8 were compared between wild-type and the mutant strains mentioned above, focusing on enzymes of PPP (listed on Table S2) and correlated with the production of mycosporines from these strains. The original data was obtained from supplementary material of previous works [39,40].

### 3.6. Protein Extraction

Proteins were extracted as previously described [39,40]. Briefly, each cell pellet was treated with 1 volume of lysis buffer (100 mM sodium bicarbonate, 0.5% Triton X100, 1 mM PMSF, 2% protease inhibition cocktail (Promega, Madison, WI, USA), 2 mM TCEP) and glass beads (0.5 mm). Seven cycles of disruption of 30 s each were performed using the “cell grinder” Mini-Beadbeater-16 (Biospec, Bartlesville, OK, USA). Between each disruption cycle, the samples were incubated on ice for 1 min. Then, centrifugation was performed at 4 °C for 20 min at 14,000 rpm, and the supernatant was recovered. The supernatants were incubated for 30 min at room temperature in a 10% *v*/*v* DNase-RNase solution (0.5 M Tris–HCl (pH 7.0), 0.5 M MgCl_2_, 100 μg/mL RNase A (Boehringer Mannheim, Germany) containing 2 μL of DNase I (Boehringer Mannheim), and the final volume was adjusted to 2.5 mL with deionized water. The proteins were analyzed in acrylamide gels under denaturant conditions (SDS–PAGE) and quantified using a Pierce^®^ BCA Protein Assay Kit (Thermo Scientific, Waltham, MA, USA). The protein extracts obtained from three biological replicates (different independent cultures) were stored at −80 °C.

## Data Availability

All relevant data are included in the article and its supplemental files. The sequences of MYC cluster for all strains analyzed in this work were deposited in Genbank and listed in Table S1.

## Supplementary Materials

The supporting information can be downloaded at: https://www.mdpi.com/article/10.3390/ijms24065930/s1. References [29,30,40,62] are cited in the Table S1A.
