# Supplementary material for: Unraveling the Molecular Basis of Mycosporine Biosynthesis in Fungi
Source: Int J Mol Sci. 2023 Mar 21;24(6):5930. doi: 10.3390/ijms24065930 (PMC10057719; doi:10.3390/ijms24065930)
Supplement: Supplementary file 1 [file ijms-24-05930-s001.zip › Figure-S1.pdf]

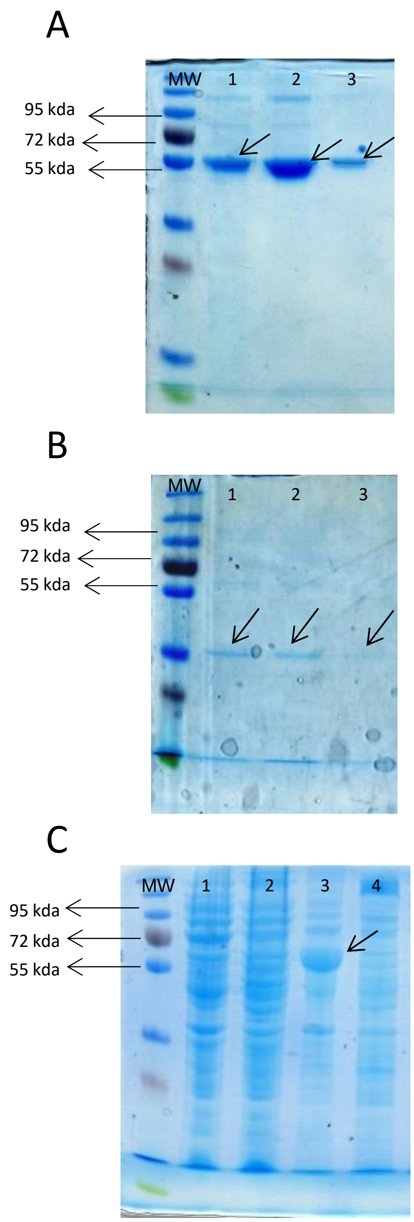

Figure S1.
SDS-PAGE of the demethyl 4-deoxygadusol synthase (DDGS), O-methyl transferase (OMT) and ATP-grasp ligase (ATPG) proteins.
(A) DDGS protein, obtained from the *E. coli* BL21(DE3) strain carrying the pET-TEV-DDGS plasmid. Lanes 1, 2 and 3: 30 µl samples of fractions 1, 2 and 3 obtained from a Histrap FF crude column (GE Healthcare), respectively. (B) OMT protein, obtained from the *E. coli* Origami 2 (DE3) strain carrying the pGB1 fox p1.OMT plasmid. Lanes 1, 2 and 3: 30 µl samples of fractions 1, 2 and 3 obtained from a Histrap FF crude column (GE Healthcare), respectively. (C) Total protein samples obtained from *E. coli* BL21 (DE3) carrying the pET-TEV-ATPG plasmid. Lanes 1 and 2, soluble fractions induced and not induced with IPTG. Lanes 3 and 4, insoluble fractions induced and not induced, respectively. Arrows indicate the positions of the DDGS, OMT and ATPG proteins. Lane MW, molecular weight standard.
