# Supplementary material for: Unraveling the Molecular Basis of Mycosporine Biosynthesis in Fungi
Source: Int J Mol Sci. 2023 Mar 21;24(6):5930. doi: 10.3390/ijms24065930 (PMC10057719; doi:10.3390/ijms24065930)
Supplement: Supplementary file 1 [file ijms-24-05930-s001.zip › Figure-S2.pdf]

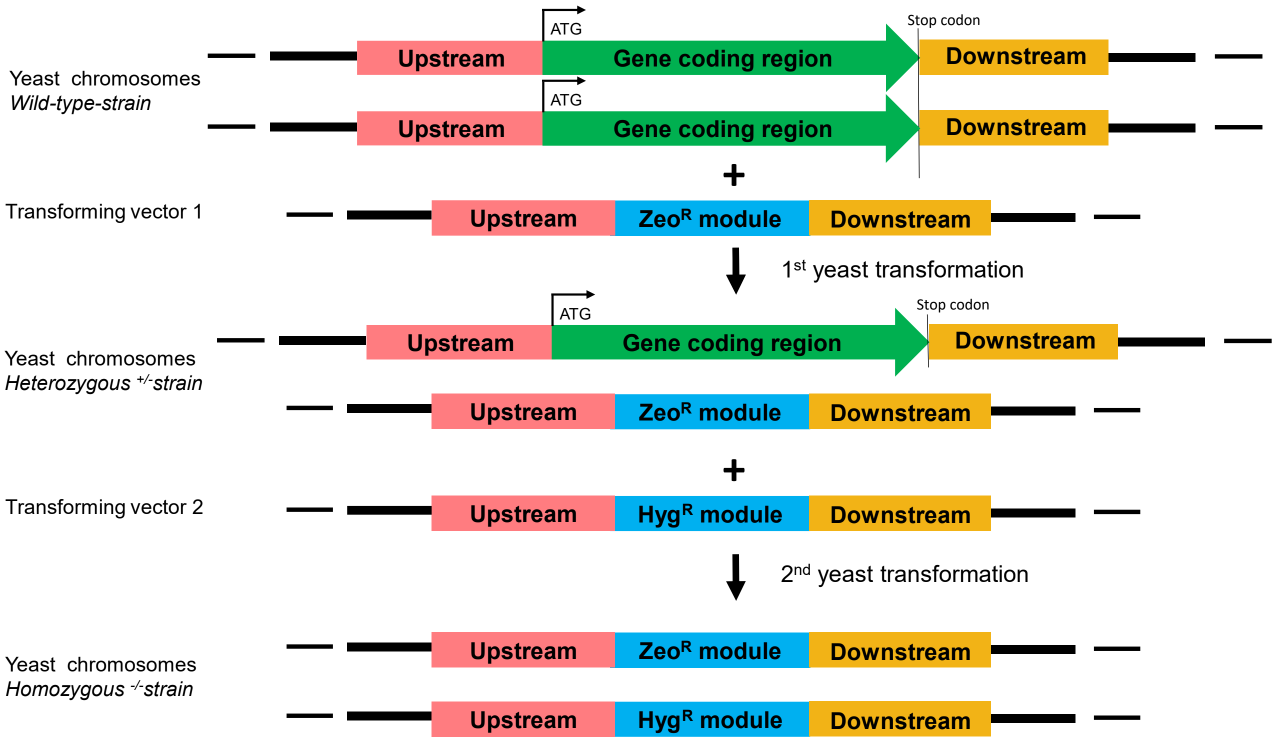

Figure S2. Scheme for homozygous mutant construction in *P. rhodozyma*. The diploid wild-type strain UCD 67-385 was transformed with a zeocin resistance module to obtain the heterozygous transformant (*GENE*$^{+}$/Δ*gene*$^{-}$) by homologous recombination. Then, this strain was transformed with a hygromycin resistance module to obtain the homozygous null mutant Δ*gene*−/−, which was able to grow in selective medium with both antibiotics. The upstream and downstream regions of single, double or triple target genes were specific for *DDGS*, *OMT*, *ATPG*, DDGS-OMT, OMT-ATPG and DDGS-OMT-ATPG in this yeast and allowed the removal of the complete gene coding region.
