# Supplementary material for: Unraveling the Molecular Basis of Mycosporine Biosynthesis in Fungi
Source: Int J Mol Sci. 2023 Mar 21;24(6):5930. doi: 10.3390/ijms24065930 (PMC10057719; doi:10.3390/ijms24065930)
Supplement: Supplementary file 1 [file ijms-24-05930-s001.zip › Figure-S3.pdf]

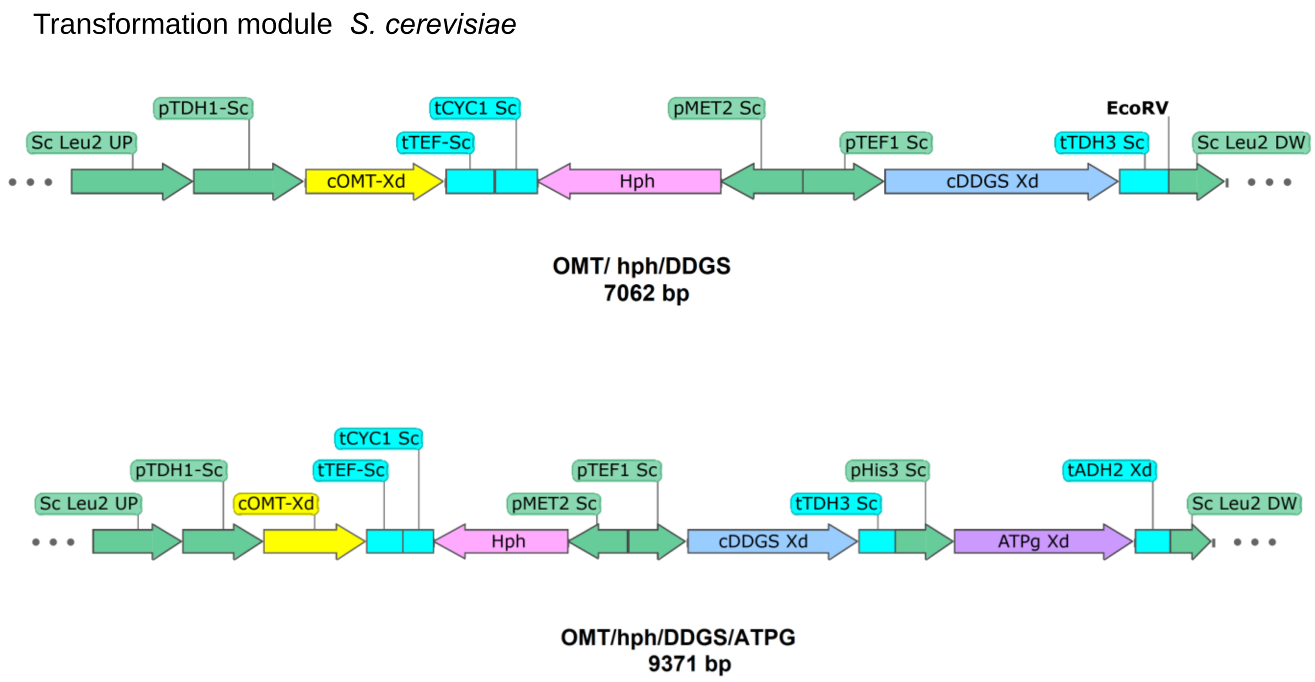

Figure S3. Yeast transformation modules for heterologous expression of *P. rhodozyma* mycosporinogenic genes in *S. cerevisiae*. (A) OMT/hph/DDGS module for 4-deoxygadusol production. (B) OMT/hph/DDGS/ATPG module for mycosporine glutaminol production. Both modules were designed to integrate into the LEU2 locus of chromosome III of *S. cerevisiae*, disrupting it. The respective Sc_OMT/hph/DDGS and Sc_OMT/hph/DDGS/ATPG strains are auxotrophic for leucine.
