# Supplementary material for: Unraveling the Molecular Basis of Mycosporine Biosynthesis in Fungi
Source: Int J Mol Sci. 2023 Mar 21;24(6):5930. doi: 10.3390/ijms24065930 (PMC10057719; doi:10.3390/ijms24065930)
Supplement: Supplementary file 1 [file ijms-24-05930-s001.zip › Figure-S4.pdf]

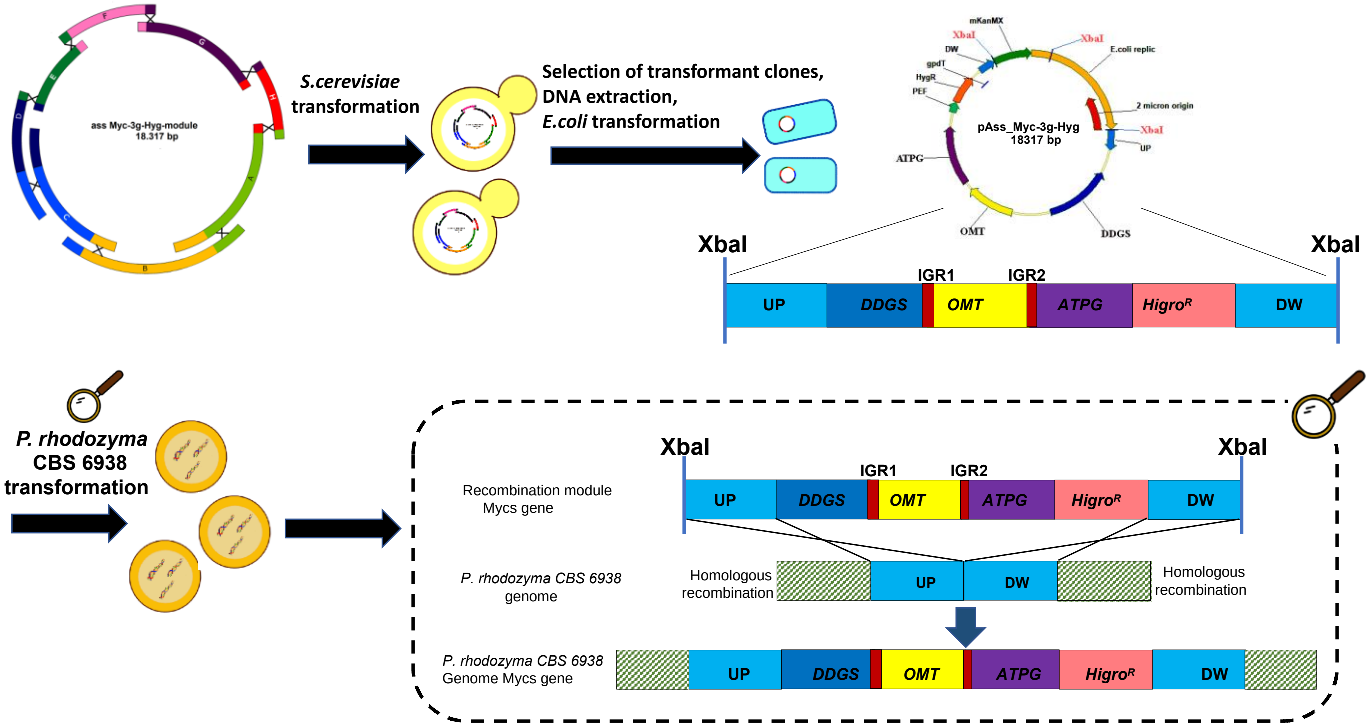

Figure S4. Plasmid pAss_Myc-3g-Hyg construction by DNA assembler and homologous recombination in *S. cerevisiae* to obtain the CBS-Myc module to transform and express MYC genes in the nonmycosporinogenic CBS 6938 strain of *P. rhodozyma*. To obtain the strain CBS 6938_MYC, eight DNA fragments (Table S1 G) with complementary ends were PCR amplified and used to transform the S288C strain of *S. cerevisiae* to allow their assembly in vivo through homologous recombination. *S. cerevisiae* transformants were selected by their resistance to G-418. The total DNA of the positive clones was used to transform *E. coli* DH-5α by electroporation. Positive *E. coli* clones were selected by ampicillin resistance, and the presence of the MYC cluster was observed by PCR. DNA of the pAss_Myc-3g-Hyg plasmid was purified and digested with *Xba*I to release the Ass_Myc-3g-Hyg recombination module, which was used to transform *P. rhodozyma* CBS 6938. The expected double homologous recombination event to generate the transformed strain is shown enclosed in a dotted line [36,37].
