# Supplementary material for: Unraveling the Molecular Basis of Mycosporine Biosynthesis in Fungi
Source: Int J Mol Sci. 2023 Mar 21;24(6):5930. doi: 10.3390/ijms24065930 (PMC10057719; doi:10.3390/ijms24065930)
Supplement: Supplementary file 1 [file ijms-24-05930-s001.zip › Figure-S5.pdf]

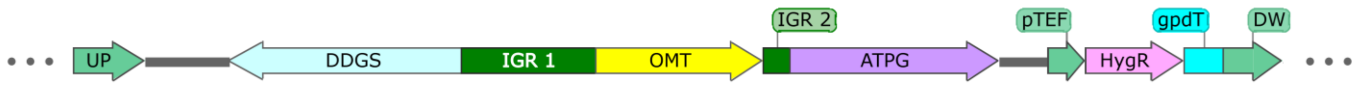

Ass_Myc-3g-Hyg
12571 bp

Figure S5. Integration of the Ass-Myc-3g-Hyg module at the end of Uchile_Xden1. PacBionly_10.1 of 1107 kb of the strain CBS 6938 of *P. rhodozyma*. The receptor wild-type strain CBS 6938 was transformed with the hygromycin resistance module Ass_Myc-3g-Hyg to obtain the transformant CBS 6938_MYC by homologous recombination.
