# Supplementary material for: Unraveling the Molecular Basis of Mycosporine Biosynthesis in Fungi
Source: Int J Mol Sci. 2023 Mar 21;24(6):5930. doi: 10.3390/ijms24065930 (PMC10057719; doi:10.3390/ijms24065930)
Supplement: Supplementary file 1 [file ijms-24-05930-s001.zip › Table S1.pdf]

Supplementary Table S1. Material and Methods

A. Yeast strains used in this work

Strain	Description	Reference/source
E. coli DH5α	F- Φ80d lacZΔM15Δ(lacZY-argF)U169 deoR recA1 endA1 hsdR17(rk- mk+) phoA supE44l- thi-1 gyrA96 relA1	[62]
S. cerevisiae S288C	Haploid wild-type strain.<br>Non mycosporin-producing strain	ATCC 204508
Sc_OMT/hph/DDGS	*S. cerevisiae* S288C Hyg^r strain containing the cDNA of the *P. rhodozyma DDGS*, and *OMT* genes and *E. coli* Hyg^r *hph* gene in the locus *LEU2* on the chromosome III, obtained by transformation of the S288C strain with the module (OMT/hph/DDGS).<br>4-deoxygadusol-producing strain.	This work
Sc_OMT/hph/DDGS/ATPG	*S. cerevisiae* S288C Hyg^r strain containing the cDNA of the *P. rhodozyma DDGS*, *OMT* and *ATPG* genes and *E. coli* Hyg^r *hph* gene in the locus *LEU2* on the chromosome III, obtained by transformation of the S288C strain with the module (OMT/hph/DDGS/ATPG).<br>Mycosporin glutaminol-producing strains.	This work
P. rhodozyma CBS 6938	Wild-type strain (Hyg^S and Zeo^S).<br>Non mycosporin-producing pigmented strain	ATCC96594
CBS 6938_MYCs	Strain CBS 6938 containing the cluster with the three mycosporine genes (MYC Cluster) of strain UCD 67-385 at the end of Uchile_Xden1-PacBionly_10 contig. Mycosporine glutaminol glucoside-producing pigmented strain	This work
P. rhodozyma UCD 67–385	Diploid wild-type strain (Hyg^S and Zeo^S).<br>Mycosporine glutaminol glucoside-producing pigmented strain.	ATCC 24230
Δ*ddgs*$^{-/-}$	Homozygous mutant gene *ddgs*_Zeo^r_Hyg^r (*ddgs*::*ble*/*ddgs*:: *hph*) strain, obtained by transformation of the mutant strain heterozygous gene *DDGS* Zeo (*DDGS/ddgs*::*ble*) with the module Δ*ddgs*:: *hph* . Pigmentation like wild strain.	This work
Δ*omt*$^{-/-}$	Homozygous mutant gene *OMT_* Zeo^r_Hyg^r (*omt*::*ble*/*omt*:: *hph*), obtained by transformation of the mutant strain heterozygous gene *omt* Zeo (*OMT1/omt*::*hph*) with the module Δ*omt*:: *hph*.<br>Redder pigmentation than wild strain.	This work
Δ*atpg*$^{-/-}$	Homozygous mutant gene *ATPG_* Zeo^r_Hyg^r (*atpg*:*ble*/*atpg*:: *hph*), obtained by recombination of the mutant strain heterozygous gene *atpg* Zeo^r (*atpg::ble/atpg*::*ble*).<br>Redder pigmentation than wild strain.	This work
Δ(*ddgs_omt)* $^{-/-}$	Homocygous double mutant genes *DDGS_OMT* Zeo^r_Hyg^r (*ddgs_omt*::*ble*/*ddgs_omt*:: *hph*), obtained by transformation of the mutant strain heterozygous genes *DDGS_OMT* Zeo^r (*DDGS_OMT/ddgs_omt*::*ble*) with the module	This work

	Δ(*ddgs_omt)*:: *hph* . Pigmentation like wild strain	
Δ(*omt_atpg)* $^{-/-}$	Homocygous double mutant genes *OMT_ATPG* Zeo_hyg (*omt_atpg)*::*ble*/(*omt_atpg)*:: *hph*), obtained by transformation of the mutant strain heterozygous genes *OMT_ATPG* Zeo^{r} (*OMT_ATPG/omt_atpg*::hph) with the module Δ(*omt_atpg)*:: *hph* . Pigmentation like wild strain.	This work
Δ(*ddgs_omt_atpg)* $^{-/-}$	Homocygous triple mutant genes *DDGS_OMT_ATPG* Zeo^{r}_hyg^{r} (ddgs_*omt_atpg)*::*ble*/(ddgs_*omt_atpg)*:: *hph*), obtained by transformation of the mutant strain heterozygous genes *DDGS_OMT_ATPG* Zeo^{r} (*DDGS_OMT_ATPG/ddgs_omt_atpg*::hph) with the module Δ(*ddgs_omt_atpg)*:: *hph* . Pigmentation like wild strain.	This work
Δ*arom*$^{-/-}$	Homozygous mutant gene *AROM*_Zeo^{r}_hyg^{r} (arom::*ble*/*arom*:: *hph*), obtained by transformation of the mutant strain heterozygous gene *AROM* Zeo^{r} AROM/*arom*::*ble*) with the module Δ*arom*:: *hph* . Pigmentation like wild strain.	This work
Δs*kn7*$^{-/-}$; Δ*rox1*$^{-/-}$ y Δ*yap6*$^{-/-}$	Homozygous mutant gene *SKN7*_Zeo^{r}_hyg^{r} (*skn7*::*ble*/*skn7*:: *hph*), *ROX1*_Zeo^{r}_hyg^{r} (*rox1*::*ble*/*rox1*:: *hph*) and *YAP6*_Zeo^{r}_Zeo^{r} (*yap6g*::*ble*/*yap6*:: *ble*) respectively.	[40]
Δ*mig1*$^{-/-}$	Homozygous mutant gene *MIG1*_Zeo^{r}_hyg^{r} (*mig1*::*ble*/mig1:: *hph*).	[29]
Δ*cyc8*$^{-/-}$ and Δ*tup1*$^{-/-}$	Homozygous mutant of genes *CYC8*_Zeo^{r}_hyg^{r} (*cyc8*::*ble*/*cyc8*:: *hph*) and *TUP1*_Zeo^{r}_hyg^{r} (*tup1*::*ble*/*tup1*:: *hph*) respectively.	[30]

ATCC: American Type Culture Collection

B. Deletion modules of mycosporine genes of UCD 67-385 strain.

Name	Deleted gen	Integration Up pb	Integration Down pb	Deletion size pb
del-*DDGS*	*DDGS*	726	827	2414
del-*OMT*	*OMT*	725	705	1361
del-ATPG	*ATPG*	664	821	1909
del-*DDGS_OMT*	*DDGS - OMT*	726	712	5343
del-*OMT_ATPG*	*OMT - ATPG*	743	821	3891
del-*DDGS_OMT_ATPG*	*DDGS-OMT-ATPG*	855	897	7873
Del-*AROM*	*AROM*	868	904	5456

C. Plasmids constructed and employed

Plasmids	Description	Reference/source
pBluescript SK-	pBluescript SK-ColE1 ori; AmpR; cloning vector with blue-white selection. Stratagene (pBS)	Stratagene
pIR-zeo	pBS contained at the *Eco*RV site a cassette of 1,2 kb bearing the *Streptoalloteichus hindustanus* Zeocin resistance *Sh ble* gene under the EF-1 α promoter and GPD transcription terminator of *X. dendrorhous*	[48]
pMN-*hph*	pBS contained at the *Eco*RV site a cassette of 1,8 kb bearing the *E. coli*-hygromicin B resistance (*hph*) gene under EF-1 a promotor and the *GPD* transcription terminator of *X. dendrorhous.*	[60]
p∆DDGS-Xd::ble	pBS contained at the *Eco*RV, 726 pb upstream, 827 pb downstream of the *DDGS* gene and the Zeocin resistance cassette between them.	This work
p∆DDGS-Xd::hph	pBS contained at the *Eco*RV, 726 pb upstream, 827 pb downstream of the *DDGS* gene and the Hygromicin B resistance cassette between them.	This work
p∆OMT-Xd::ble	pBS contained at the *Eco*RV, 725 pb upstream, 705 pb downstream of the *OMT* gene and the Zeocin resistance cassette between them.	This work
p∆OMT-Xd::hph	pBS contained at the *Eco*RV, 725 pb upstream, 705 pb downstream of the *OMT* gene and the Hygromicin B resistance cassette between them.	This work
p∆ATPG-Xd::ble	pBS contained at the *Eco*RV, 664 pb upstream, 821 pb downstream of the *ATPG* gene and the Zeocin resistance cassette between them.	This work
p∆ATPG-Xd::hph	pBS contained at the *Eco*RV, 664 pb upstream, 821 pb downstream of the *ATPG* gene and the Hygromicin B resistance cassette between them.	This work
p∆(DDGS-OMT)Xd::ble	pBS contained at the *Eco*RV, 726 pb upstream, 712 pb downstream of the (*DDGS-OMT*) genes and the Zeocin resistance cassette between them.	This work
p∆(DDGS-OMT)Xd::hph	pBS contained at the *Eco*RV, 726 pb upstream, 712 pb downstream of the the (*DDGS-OMT*) genes and the Hygromicin B resistance cassette between them.	This work
p∆(OMT-ATPG)Xd::ble	pBS contained at the *Eco*RV, 743 pb upstream, 821 pb downstream of the *(OMT-ATPG*) genes and the Zeocin resistance cassette between them.	This work
p∆(OMT-ATPG)Xd::hph	pBS contained at the *Eco*RV, 743 pb upstream, 821 pb downstream of the *(OMT-ATPG*) genes and the Hygromicin B resistance cassette between them.	This work
p∆(DDGS-OMT-ATPG)Xd::ble	pBS contained at the *Eco*RV, 855 pb upstream, 897 pb downstream of the *(DDGS-OMT-ATPG*) genes and the Zeocin resistance cassette between them.	This work
p∆(DDGS-OMT-ATPG)Xd::hph	pBS contained at the *Eco*RV, 855 pb upstream, 897 pb downstream of the *(DDGS-OMT-ATPG*) genes and the Hygromicin B resistance cassette between them.	This work
p∆Arom-Xd::ble	pBS contained at the *Eco*RV, 868 pb upstream, 904 pb downstream of the *AROM* gene and the Zeocin resistance cassette between them.	This work
p∆Arom-Xd::hph	pBS contained at the *Eco*RV, 868 pb upstream, 904 pb downstream of the *AROM* gene and the Hygromicin B resistance cassette between them.	This work

pBS-Sc_LEU2	pBluescript SK- containing, in the *Eco*RV site, a 1095 bp DNA fragment with the *S. cerevisiae LEU2* gene. The latter also had an *Eco*RV site near the middle of its sequence.	This work
pBS-P$_{met2}$_*hph*_T_{cyc1}	pBluescript SK- containing, at the *Eco*RV site a module Hyg^{r} of 1.8 kb bearing the *E. coli hph* gene under *MET2* promoter and the *CYC1* transcription terminator of *S. cerevisiae*.	This work
pBS-P$_{Tef}$*cDDGS*_T_{TDH3}	pBluescript SK- containing, at the *Eco*RV site, a module of 2.234 kb bearing the cDNA of the *DDGS of P. rhodozyma* gene under EF-1α promoter and the *CYC1* transcription terminator of *S. cerevisiae*.	This work
pBS-P$_{TDH3}$OMT_T$_{tef}$	pBluescript SK- containing, at the *Eco*RV site a modulo of 1834 bp containing the *OMT of P. rhodozyma* gene under *TDH3* promoter and the EF-1 α transcription terminator of *S. cerevisiae*	This work
pBS-P$_{His3}$*ATPG*_t$_{ADH2}$	pBluescript SK- containing, at the *Eco*RV site a module of 2303 bp containing the *ATPG* of *P. rhodozyma* gene under *HIS3* promoter and the *ADH2* transcription terminator of *S. cerevisiae*	This work
pBS_OMT-DDGS	pBS-P$_{Tef}$*cDDGS*_T_{TDH3} containing on the *Sma*I site the Sc_ P$_{TDH3}$OMT_T$_{tef}$ module from pBS-P$_{TDH3}$OMT_T$_{tef}$.	This work
pBS_OMThphDDGS	pBS_OMT-DDGS containing on the *Eco*RI site the hygromycin resistance module (Sc_P$_{met2}$_*hph*_T_{cyc1}).	This work
p*Sc_OMThphDDGS*	*pBS-Sc_LEU2* containing, at the *Eco*RV on *LEU2* gene, the DNA module bearing the cDNA of the *P. rhodozyma* c*OMT* and *cDDGS*, genes and *E. coli* Hyg^{r} *hph* gene under *S. cerevisiae* Promoters and transcription terminators.	This work
pSc_*OMT/hph/DDGS/ATPG*	*pSc_OMThphDDGS* containing, at a new *Eco*RV site between the end of transcription T$_{TDH3}$ and the LEU2 down middle gene, the ATPG module from pBS-P$_{His3}$*ATPG*_t$_{ADH2}$ plasmid under *S. cerevisiae* Promoters and transcription terminators.	This work
pAss-Myc-3g-Hyg-to-CBS	pBluescript SK- containing the 2μ ORI and G418 resistance gene flanked by the CBS 6938 recombination region (side A and B) bearing a *Xba*I restriction site on each end, followed by the MYC cluster and the hygromycin resistance module.	This work

D. Primers used for the construction of deletion mutants of *P. rhodozyma* modules.

TEF-Xd_antisense	TCC TAA TTC TTG TCG ACA ACG
gpd_Xd-sense	GGA CAA GGC AAG AAG TGA GCA
Hyg-seq-Fw	GTA TAT GCT CCG CAT TGG TCT
Zeoc-sense-Fw	ACG ACG TGA CCC TGT TCA TCA
Zeoc-antisense-Rv	TGA TGA ACA GGG TCA CGT CGT
Hyg-Seq-RV	CGA TGC AAA GTG CCG ATA AAC
Pre_DDGS-del-Rv	ACG TAA GAA CAG AAC GAC TTG GG
gDDGS-del-Fw2	GGT CAC CGG AAA CGG ATC TTA
gDDGS-del-HpaI-Rv2	TCA TCA AAA ACT GTT AAC TTT CAA CTC CGA AGT AAC G
gDDGS-del-HpaI-Fw2	CGG AGT TGA AAG TTA ACA GTT TTT GAT GAA CGC AGA T
gDDGS-del-Rv2	AAT CCG TGC AGA CCA TGT TAC

gOMT-del-Fw1	ATC AGG ATC GTG CTT GAA TCG CT
gOMT-del-HpaI-Rv1	TGA GCA TCT GGT TAA CAA TAA AGC TTA TGA TCA AAG G
gOMT-del-HpaI-Fw1	TAA GCT TTA TTG TTA ACC AGA TGC TCA TTT TCA TGA TC
gOMT-del-Rv1	TTG CTC ATC GCT CTA GCT ACG
ATPG-del-fw1	TAC ACA GCT GAA GAG GAC GCG TT
ATPG-del-HpaI-Rv1	TGA CAC AGT ATG TTA ACG CCA AGT CCA ATA AGA TGT AG
ATPG-del-Hpa-Fw1	TTG GAC TTG GCG TTA ACA TAC TGT GTC AAC AAT CAT AAC
ATPG-del-Rv1	GAC TTG GGC TGT CGG ATG AGA TA
Del_DDGS-OMT_HpaI-Rv	TCT GAA AAG TGG TTA ACT TTC AAC TCC GAA GAT AAC G
Del_DDGS-OMT_HpaI-Fw	CGG AGT TGA AAG TTA ACC ACT TTT CAG ATG CTC ATT T
Del_OMT-Agrasp_HpaI-Fw	TCA ACC CTC GGT TAA CAT ACT GTG TCA ACA ATC ATA AC
Del_OMT-ATPgrasp_HpaI-Rv	GAC ACA GTA TGT TAA CCG AGG GTT GAT GAA ACC AAA T
post-gATPG-Rv	TTC CGT GGC ACC GAA GAT TG
pre-gOMT-Fw	GGT CCC GGT CAA ACT GAA TGT
gArom-fw	GTC CGC TTG ATC CCT TGA TCT
gArom-Rv	TCG GAA CTG CAA AGT GTA TCC
gArom-del-HpaI-fw	TGT TCA GGA CCG TTA ACC TGG AAG GAT TGC CGT CCT T
gArom-del-HpaI-Rv	AAT CCT TCC AGG TTA ACG GTC CTG AAC AGA TGA GAC C
pre-gArom-Fw	AGG AAC CTC TCT TCT TAC CGA G
Post-gArom-Rv	TTA CAG TCC ATC TCT ACG GGC
cTR-Arom-Fw	AGC CGC ATC TAT CTC GAG TCA
cTR-Arom-Rv	CTC CTC GCA TTC CGA TTA TG
cATPG_Fw-b	ATG CCA TCC CTA CTA CCA TCT G
cATPG_Rv	CTA ACA CTC AAA TAC CCG AGC CGT
cOMT_Fw	ATG TCT TCC ACC ACA AAT CAC
COMT_Rv	TTA TTG CTC CAG TCC CTT GCA AAC
cOMT_Fw	ATG TCT TCC ACC ACA AAT CAC
COMT_Rv	TTA TTG CTC CAG TCC CTT GCA AAC
cDHQS-Fw	ATG TGT GAC TGC AAC AAC ATC
cDHQS-Rv	TTA AAG AGA CTC GAC AGA AGA

E. Primers used for the construction of S. cerevisiae mycosporine expression module.

Hyg-seq-Fw	GTA TAT GCT CCG CAT TGG TCT
pAdh1-hyg-Rv1	CGA TGC AAA GTG CCG ATA AAC
pAdh1-hyg-Rv1	CAG GCT TTT TCA TTG TAT ATG AGA TAG TTG ATT
Hyg-pAdh1-Fw1	TAT CTC ATA TAC AAT GAA AAA GCC TGA ACT CAC
pMet2-hyg-Rv1	CAG GCT TTT TCA TTC CTT TTA TAC TAC GTT TTT
Hyg-pMet2-Fw1	TAG TAT AAA AGG AAT GAA AAA GCC TGA ACT CAC
pMET2-Fw	GCC TCT GAG CAG TAT AAA TTG
tCYC1-RV	AGC TTG CAA ATT AAA GCC TTC G
pTDH3-Sc-Fw	AGT TCG AGT TTA TCA TTA TCA
tTEF_Sc-Rv	AAA AGA CCA AAC GGT GAC GTT AAG
pTDH3-Sc-HpaI-Rv	TCT CCG TTA ACT TTG TTT GTT TAT GTG TGT TTA TTC G
tTEF-Sc-HpaI-Fw	AAA CAA ACA AAG TTA ACG GAG ATT GAT AAG ACT TTT C
pHis3-Sc-Fw	TTC TCG ACG TGG GCC TTT TTC
tADH2-Sc-HpaI-Fw	AAG CAA CAG GCG CGT TGG AC
pTEF_Sc-HpaI-Rv	GTG TTC GCA AAG TTA ACT TTG TAA TTA AAA CTT AGA T
tTDH3-HpaI-Fw	TAA TTA CAA AGT TAA CGT GAA TTT ACT TTA AAT CTT G
pTEF-HpaI-Rv	GTA AAT TCA CGT TAA CTT TGT AAT TAA AAC TTA GAT T

F. Primers used for the construction of the fragments components of CBS 6938 mycosporine expression module.

2micSc-MicA-Fw	TACTTTTGAGCAATGTTTGTGGAAGCGGTATTCGCAATGTCTAGACGTGATGGCAAGATC
MicA-2micSc-Rv	CGATCGAGCTGGATATACGGATCTTGCCATCACGTCTAGACATTGCGAATACCGCTTCCA
TEF-tMicA-Rv	AGAGCTTGTGTCGGATGAACTGTCGGCTGATGAGCCGATATTAATCATTCGTCTCACCTG
tMicA-TEF-Fw	TGTGTGAAGTCTTGCACTACAGGTGAGACGAATGATTAATATCGGCTCATCAGCCGACAG
gpd-Rec_CBS-Rv	AAGATCATAAAGAGAGGAAGTCGGCGAATGAAGTGAGCTTATCATGAGAGATGACGGAGA
Rec_CBS-gpd-F	ATCTGTTGACCATCACCATCATCTCCGTCATCTCTCATGATAAGCTCACTTCATTCGCCG
Kn-Rec_CBS-Rv	AAGACTGTCAAGGAGGGTATTCTGGGCCTCCATGTCTCTAGAACCCTTTCGAAACGAAAC
Rec_CBS-Kn-Fw	GGATCAGTAGGGGTTATGTTTCGTTTCGAAAGGGTTCTAGAGACATGGAGGCCCAGAATA

G. DNA fragments components used to obtain the plasmid pAss-Myc-3g-Hyg-to-CBS by the assembly method in *S. cerevisiae* S288C for the construction of CBS 6938 mycosporine expression module.

Name	Color	Size (bp)	Components
A	Green	4107	DNA 2μ of *S. cerevisiae* and partial *DDGS* gene
B	Orange	4391	Partial *DDGS* gene - partial *OMT* gene
C	Dark blue	4054	Partial OMT gene and part of ATPG gene
D	Light blue	2935	Part of ATPG gene and part of Hyg^r module.
E	Dark green	1900	Part of Hyg^r resistance module, Part of region DW of recombination.
F	Pink	2622	Part of region DW, part of G418 resistance gene (Kan^r).
G	Violet	3231	Part of G418 resistance gene and partial pBluescript vector.
H	Red	1585	Partial pBluescript and 2μ DNA of *S. cerevisiae*.

H. Structural organization of the Cluster of Mycosporine genes of the species of the *Phaffia* genera used in this work.

		Size of genes and sequences (bp)						
Specie	Strain	*DDGS*	*IGR1*	*OMT*	*IGR2*	*ATPG*	Cluster	GenBank
P. rhodozyma	UDC 67-385	2414	1568	1361	621	1909	7873	OQ547787
	AVHN2	2405	1565	1370	617	1912	7869	OQ547788
	VOH	2405	1565	1370	616	1912	7868	OQ547789
	RV4	2401	1563	1370	616	1912	7862	OQ547790
	RV7.6	2401	1563	1370	616	1912	7862	OQ547791
	RV7.40	2401	1563	1370	616	1912	7862	OQ547792
	CRUB1149	2402	1565	1370	616	1912	7865	BankIt2677958
	CBS 7918	2422	1569	1362	622	1909	7884	BankIt2677971
P. australis	CBS14095	2379	1568	1365	611	1904	7827	BankIt2679089
P. tasmanica	CBS14096	2376	1544	1360	519	1958	7757	BankIt2679147
P. brasiliana	Y6497	2386	1573	1333	573	1907	7772	BankIt2678417
	Y6498	2386	1573	1333	573	1907	7772	BankIt2678800
P. aurantiaca	CBS15548	2377	1559	1361	616	1912	7825	BankIt2679178
A. fabae	247/15	1554	1432	965	744	1567	6262	BankIt2679542
