# Supplementary material for: Unraveling the Molecular Basis of Mycosporine Biosynthesis in Fungi
Source: Int J Mol Sci. 2023 Mar 21;24(6):5930. doi: 10.3390/ijms24065930 (PMC10057719; doi:10.3390/ijms24065930)
Supplement: Supplementary file 1 [file ijms-24-05930-s001.zip › Table S2.pdf]

Supplementary Table S2. Proteomic and transcriptomic data of mycosporine-related genes in various mutant strains from previous studies.

Strain	Condition	Source	Log_2(mut/wt)	FDR *p-value*	Reference
**Phosphogluconate dehydrogenase (*PGD*, K00033*)**					
Δ*cyc8*$^{-/-}$	Minimal medium MMv supplemented with glucose 2%, early exponential growth phase.	Proteomic	0.58	0.003	[39]
Δ*tup1*$^{-/-}$	Minimal medium MMv supplemented with glucose 2%, early exponential growth phase.	Proteomic	0.48	0.023	[39]
Δ*rox1*$^{-/-}$	Minimal medium YNB supplemented with glucose 2%, early exponential growth phase.	Proteomic	-0.27	0.000	[40]
Δ*yap6*$^{-/-}$	Minimal medium YNB supplemented with glucose 2%, early exponential growth phase.	Transcriptomic	-0,76	0.001	[40]
**Phosphogluconolactonase (*PGL*, K01057*)**					
Δ*skn7*$^{-/-}$	Minimal medium YNB supplemented with glucose 2%, early exponential growth phase.	Transcriptomic	-0.90	0.000	[40]
Δ*yap6*$^{-/-}$	Minimal medium YNB supplemented with glucose 2%, early exponential growth phase.	Transcriptomic	-0.63	0.018	[40]
**Transaldolase (*TAL*, K00616*)**					
Δ*mig1*$^{-/-}$	Minimal medium MMv supplemented with glucose 2%, early exponential growth phase.	Proteomic	-0.56	0.009	[39]
Δ*skn7*$^{-/-}$	Minimal medium YNB supplemented with glucose 2%, early exponential growth phase.	Proteomic	0.79	0.000	[40]
Δ*yap6*$^{-/-}$	Minimal medium YNB supplemented with glucose 2%, early exponential growth phase.	Proteomic	0.47	0.000	[40]

Ribokinase (*RBK*, K00852*)

Δ*skn7*$^{-/-}$	Minimal medium YNB supplemented with glucose 2%, early exponential growth phase.	Transcriptomic	0.72	0.067	[40]
Δ*yap6*$^{-/-}$	Minimal medium YNB supplemented with glucose 2%, early exponential growth phase.	Proteomic	1.68	0.015	[40]

Xylulose phosphoketolase (*XPK*, K01621*)

Δ*rox1*$^{-/-}$	Minimal medium YNB supplemented with glucose 2%, early exponential growth phase.	Proteomic	-0.69	0.000	[40]
Δ*yap6*$^{-/-}$	Minimal medium YNB supplemented with glucose 2%, early exponential growth phase.	Proteomic	-0.48	0.000	[40]

* KEGG Orthology number (K) from each gene was provided.
